# Governance of Protecting Patients

**DOI:** 10.1192/bjo.2021.364

**Published:** 2021-06-18

**Authors:** Sureshkumar Bhatt

**Affiliations:** St. Tammany Parish Coroners Office

## Abstract

**Aims:**

Involuntary commitment is a legal process through which an individual with symptoms of severe mental illness is court-ordered into inpatient or outpatient treatment. These criteria vary between nations. The goal of this presentation is to compare the governance of protecting patients among different parts of the world.

**Background:**

Understanding the relevance of the judicial committeemen in psychiatry is an essential part of good psychiatric practice. A majority of patients who need inpatient psychiatric treatment fall into one of the following categories: dangerous to self, dangerous to others, or gravely disabled.

In the United States, the Parens Patriate doctrine has had great application in the treatment of mentally ill persons, children, and other individuals who are legally incompetent to manage their affairs. The states, which act as parens patriae, can make decisions regarding mental health treatment. State law governs involuntary commitment, and procedures may vary among states.

**Method:**

One of the essential duties of St. Tammany Parish Coroner Office, Louisiana, USA is Mental Health Service, From January 2017 to October 2019, 887 Order of Protective Custody (OPC), 17,838 Physician Emergency Certificates (PEC), and 13096 Coroner Emergency Certificates (CEC) were issued. These certificates allow legal authority to transport a patient to the nearest ER for assessment by physician and mental health providers.

**Result:**

Patients with active Physician Certificate are examined by a coroner according to patient's mental history and clinical presentation. Coroner Certificate helps the treatment facilty detail the patient for diagnosis and treatment for fifteen days.

**Conclusion:**

St. Tammany Parish Coroner Office is fulfilling its responsibility to provide proper mental health to psychiatric patients. It is necessary for each country/state/parish to have legal structure and provide proper care who are dangerous to self or others, or gravely disabled. The procedures of OPC, PEC, and CEC will be presented.

